# Guar Gum Stimulates Biogenic Sulfide Production at Elevated Pressures: Implications for Shale Gas Extraction

**DOI:** 10.3389/fmicb.2017.00679

**Published:** 2017-04-19

**Authors:** Sophie L. Nixon, Leanne Walker, Matthew D. T. Streets, Bob Eden, Christopher Boothman, Kevin G. Taylor, Jonathan R. Lloyd

**Affiliations:** ^1^School of Earth and Environmental Sciences, University of ManchesterManchester, UK; ^2^Rawwater Engineering Company LimitedCulcheth, UK

**Keywords:** sulfate-reducing bacteria, guar gum, bioreactor, hydraulic fracturing, organic carbon

## Abstract

Biogenic sulfide production is a common problem in the oil industry, and can lead to costly hydrocarbon processing and corrosion of extraction infrastructure. The same phenomenon has recently been identified in shale gas extraction by hydraulic fracturing, and organic additives in fracturing fluid have been hypothesized to stimulate this process. Constraining the relative effects of the numerous organic additives on microbial metabolism *in situ* is, however, extremely challenging. Using a bespoke bioreactor system we sought to assess the potential for guar gum, the most commonly used gelling agent in fracturing fluids, to stimulate biogenic sulfide production by sulfate-reducing microorganisms at elevated pressure. Two pressurized bioreactors were fed with either sulfate-amended freshwater medium, or low-sulfate natural surface water, in addition to guar gum (0.05 w/v%) and an inoculum of sulfate-reducing bacteria for a period of 77 days. Sulfide production was observed in both bioreactors, even when the sulfate concentration was low. Analysis of 16S rRNA gene sequences indicate that heterotrophic bacteria closely associated with the genera *Brevundimonas* and *Acinetobacter* became enriched early in the bioreactor experiments, followed by an increase in relative abundance of 16S rRNA genes associated with sulfate-reducing bacteria (*Desulfosporosinus* and Desulfobacteraceae) at later time points. Results demonstrate that guar gum can stimulate acid- and sulfide-producing microorganisms at elevated pressure, and may have implications for the potential role in microbially induced corrosion during hydraulic fracturing operations. Key differences between experimental and *in situ* conditions are discussed, as well as additional sources of carbon and energy for biogenic sulfide production during shale gas extraction. Our laboratory approach can be tailored to better simulate deep subsurface conditions in order to probe the role of other fracturing fluid additives and downhole parameters on microbial metabolisms observed in these systems. Such baseline studies will prove essential for effective future development of shale gas worldwide.

## Introduction

The recent development of shale gas in the United States has dramatically increased domestic gas supply, and the UK and other European countries are planning to follow suit in the coming decades and exploit their own shale gas reserves (Andrews, 2013; Weijermars, 2013). The lead up to this development offers the opportunity to learn from and avoid problems encountered in the US, among them the biogenic production of hydrogen sulfide. This process, known as souring, causes pitting and cracking of susceptible materials (increasing the risk of leaks), can form pyrophoric films on steel in gas lines (posing a fire risk), and necessitates costly removal from produced gas. These issues can potentially lead to environmental and reputational damage, and can ultimately shorten the life span of a shale gas well (Eden et al., 1993). Biogenic hydrogen sulfide has been reported at a number of shale gas wells in the Barnett Shale (Fichter et al., 2008, 2009), and several studies have since demonstrated the presence of sulfidogenic bacteria in produced waters from this and other shale gas plays (Davis et al., 2012; Kirk et al., 2012; Struchtemeyer and Elshahed, 2012; Mohan et al., 2013a,b; Cluff et al., 2014; Akob et al., 2015).

Shale gas exists in unconnected or poorly-connected sub-micron scale spaces within very low permeability shale. It is therefore necessary to artificially fracture the formation in order to liberate shale gas. This is achieved through hydraulic fracturing, in which water-based fluids are pumped down the well at high pressure in order to overcome confining pressures and induce fractures in the formation. Wells are first drilled vertically to the required depth in the shale formation, then horizontally to increase contact with the gas-bearing rock. The fractures generated in this process are held open by a proppant, typically sand, to allow free flow of natural gas to the well. The fluid used to fracture the host rock is water-based, and includes a host of additives in addition to sand. Each serves a particular function, and the mix of additives varies from one operation to another, depending primarily on the characteristics of the source formation, though many such additives are organic (Elsner and Hoelzer, 2016). Here we address the hypothesis that organic fracturing fluid additives stimulate biogenic sulfide production.

A number of organic fracturing fluid additives are already known to be bioavailable to microorganisms. For example, ethylene glycol is widely used as a surfactant to inhibit scale formation during shale gas extraction (Elsner and Hoelzer, 2016), but is readily degraded and used for growth by soil and sewage microorganisms (Haines and Alexander, 1975; Watson and Jones, 1977; McGahey and Bouwer, 1992). Citric acid, used as a complexing agent in almost a third of disclosed hydraulic fracturing operations to date (Elsner and Hoelzer, 2016), is readily fermented by strains of *Clostridium*, yielding further bioavailable by-products such as acetate (Walther et al., 1977; Schink, 1984). Even the most conservative fracturing fluids contain an organic polymer, which serves as a gelling agent to increase viscosity and keep the proppant in suspension. The most widely used gelling agent is guar gum (Elsner and Hoelzer, 2016), a polysaccharide also common in the food industry, and fermented by intestinal bacteria (Tomlin et al., 1986; Crociani et al., 1994). It is therefore highly likely that fracturing fluid additives will stimulate microbial metabolism in shale gas extraction operations, and are in part responsible for the observed biogenic sulfide production in these systems (Fichter et al., 2008, 2009).

Tracking the metabolism of such organic amendments and their possible coupling to sulfate reduction *in situ* is, however, extremely challenging. Here we report on a laboratory-based procedure to test the potential for the widely used gelling agent, guar gum, to stimulate biogenic sulfide production in pressurized semi-continuous bioreactor experiments. We present evidence that guar gum stimulates sulfide production. The implications for biofouling in future shale gas extraction operations are discussed. This study employs an *ex situ* bioreactor approach and represents a first step in understanding the potential microbiological implications in shale gas extraction. This approach is more appropriate than conventional microcosm-based batch tests conducted in serum vials, and can be tailored to simulate the deep terrestrial subsurface. This method could therefore serve as a blueprint for similar “baseline” studies on, for example, the microbial metabolism of fracking additives, required to support effective large-scale shale gas extraction both in the United States, and more widely in regions considering exploitation of this resource.

## Materials and Methods

### Microbial Enrichment

A sulfate-reducing enrichment culture was initiated using sediment-laden water from a drinking water reservoir near Buxton, Derbyshire in the UK. Sediment-laden water was added to 90 ml sterile (121°C 20 min) Postgate B medium (described in Tanner, 1989) amended with sodium acetate as the electron donor (31 mM), pH 7, in a sterile 100 ml serum vial. The enrichment was incubated at 30°C in the dark, and was considered positive for sulfate reduction upon the accumulation of a black precipitate (iron sulfide). Sulfate reduction was evidenced by the appearance of black precipitates (presumed iron sulfide) and was used as an inoculum for bioreactor experiments after a 17 day incubation period.

### Bioreactor Design

The pressurized bioreactors are bespoke, designed and manufactured by Rawwater Engineering Company Limited. Each bioreactor comprised a 5 cm outside diameter steel tube, lined with a unplasticized polyvinyl chloride liner, grouted in place either end prior to welding end-caps in place. Within the center of each end-cap was a weldable 0.635 cm Swagelok tube fitting. The inlet of the bioreactor was connected to a Perkins-Elmer HPLC series 100 pump, supplied by a nitrogen-pressurized aspirator at 2 barg. The outlet was connected to a union-T fitting, connected to a 0–3000 psig (0–206.8 bar) pressure gauge and a 1000 psig (68.9 bar) pressure release valve, from which samples were collected. All fittings were of 316L stainless steel.

### Bioreactor Experimental Setup

Bioreactor 1 was packed with glass beads, and was fed with an influent of sulfate-amended freshwater medium. Prior to injection medium was purged with nitrogen gas to drive off dissolved oxygen. The basal medium (pH 7) contained (in grams per liter deionized water): NaHCO_3_ (2.0), NH_4_Cl (0.25), NaH_2_PO_4_.H_2_O (0.06), KCl (0.1), MgSO_4_.7H_2_O (2.0), CaSO_4_ (1.0), and 10 ml mineral mix. The mineral contained (in grams per liter deionized water): nitrilotriacetic acid (1.5), MgSO_4_.7H_2_O (6.0), NaCl (1.0), FeSO_4_.7H_2_O (0.1), CaCl_2_ (0.076), CoCl_2_ (0.054), ZnCl_2_ (0.13), CuSO_4_.5H_2_O (0.01), AlK(SO_4_)_2_.12H_2_O (0.01), H_3_BO_3_ (0.01), MaNoO_4_.2H_2_O (0.294), and NiCl_2_.6H_2_O (0.024). All constituents of the medium were added prior to autoclaving for 20 min at 121°C. Total concentration of sulfate in this medium was 15.7 mM.

Bioreactor 2 was packed with low-iron sand (Fisher Scientific, UK), and was fed with deoxygenated surface water collected from a seasonal pond on private land in Culcheth, Cheshire, UK (53°37′44.04″N, 2°29′44.05″W). This land was chosen as a source of readily available fresh surface water relevant to potential future hydraulic fracturing scenarios in the North of England. Formate, acetate, propionate, and butyrate concentrations were measured with ion chromatography (IC; Metrohm 930 Compact IC Flex, Daresbury, UK) and found to be below the detection limit of 0.1 mg/l. Sulfate, nitrate, and phosphate concentrations in this surface water were similarly measured with IC (Dionex ICS5000 Dual Channel, Hemel Hempstead, UK), and found to be 79.6 (0.83 mM), 0.19, and 0.1 mg/l, respectively. Bioreactor 2 therefore served as a “low sulfate” comparison to Bioreactor 1. The influent was purged with oxygen-free nitrogen gas for 24 h prior to injection.

The bioreactors were run at room temperature (15–25°C) and 1000 psi (68.9 bar) under batch conditions. Injections were made at a flow rate of 3 ml/min for a 30 min batch injection twice weekly (on days 1 and 4 of each week) until day 28, then once weekly, for a total of 77 days. For each bioreactor, the weekly influent injection volume was equal to the pore volume of the bioreactor, quantified with a fluorescein tracer (Sigma-Aldrich, UK) prior to initiating the experiments. The pore volume of Bioreactor 1 was 376 ml, and 562 ml for Bioreactor 2. Both bioreactors were flushed with 6× pore volumes of anaerobic reverse-osmosis water after fluorescein tracer tests, and before the first experimental injection. A 1% (v/v) inoculum from the sulfate-reducing enrichment culture was added to the influent of both bioreactors from the start of the experiment until day 28. Guar gum (0.05% w/v) was added to the influent of both bioreactors from the start of the experiment until day 63. Samples were collected for analysis from the effluent generated at each injection point.

### Analytical Methods

Total sulfide concentrations in the effluent were measured using the methylene blue assay. Samples were collected directly onto zinc acetate crystals, which reacted with the sulfide in solution to form a zinc sulfide precipitate, preserving the aqueous sulfide concentration. The sulfide was then regenerated in acid for a methylene blue colorimetric test (Fonselius et al., 1999). The methylene blue test was calibrated using the standard iodometric determination of sulfide titration method (limit of detection 0/5 mg/l total sulfide). The pH of the effluent was measured using pH indicator strips (pH range 4–10, Fisher Scientific).

### Bacterial Community Composition

Bacterial community composition was examined by extraction of DNA from 10 ml samples of the enrichment culture influent (2 days prior to the start of experiments) and effluent using the MoBio PowerLyzer^TM^ PowerSoil DNA Isolation Kit (MoBio Laboratories, Inc., Carlsbad, CA, USA). Sequencing of polymerase chain reaction (PCR) amplicons of 16S rRNA was conducted with the Illumina MiSeq platform (Illumina, San Diego, CA, USA) targeting the V4 hyper variable regions (forward primer, 515F, 5′-GTGYCAGCMGCCGCGGTAA-3′; reverse primer, 806R, 5′-GGACTACHVGGGTWTCTAAT-3′) for 2 × 150-bp paired-end sequencing (Illumina) (Caporaso et al., 2011, 2012). PCR amplification was performed using Roche FastStart High Fidelity PCR System (Roche Diagnostics Ltd, Burgess Hill, UK) in 50 μl reactions under the following conditions: initial denaturation at 95°C for 2 min, followed by 36 cycles of 95°C for 30 s, 55°C for 30 s, 72°C for 1 min, and a final extension step of 5 min at 72°C. The PCR products were purified and normalized to ∼20 ng each using the SequalPrep Normalization Kit (Fisher Scientific, Loughborough, UK). A negative PCR control was conducted in parallel to bioreactor effluent and inoculum samples, and found to be devoid of DNA. The PCR amplicons from all samples were pooled in equimolar ratios. The run was performed using a 4 pM sample library spiked with 4 pM PhiX to a final concentration of 10% following the method of Kozich et al. (2013). Raw sequences were divided into samples by barcodes (up to one mismatch was permitted) using a sequencing pipeline. Quality control and trimming was performed using Cutadapt (Martin, 2011), FastQC^1^, and Sickle (Joshi and Fass, 2011). MiSeq error correction was performed using SPADes (Nurk et al., 2013). Forward and reverse reads were incorporated into full-length sequences with Pandaseq (Masella et al., 2012). Chimeras were removed using ChimeraSlayer (Haas et al., 2011) and operational taxonomic units (OTUs) were generated with UPARSE (Edgar, 2013). OTUs were classified by Usearch (Edgar, 2010) at the 97% similarity level, and singletons were removed. Rarefaction analysis was conducted using the original detected OTUs in Qiime (Caporaso et al., 2010). Taxonomic assignment was performed by the Ribosomal Database Project (RDP) Classifier using an 80% confidence limit (Wang et al., 2007). Raw sequencing data have been submitted to NCBI Sequence Read Archive^2^ with the project accession number SRP093359.

## Results

### Bioreactor Experiments

**FIGURE 1:**
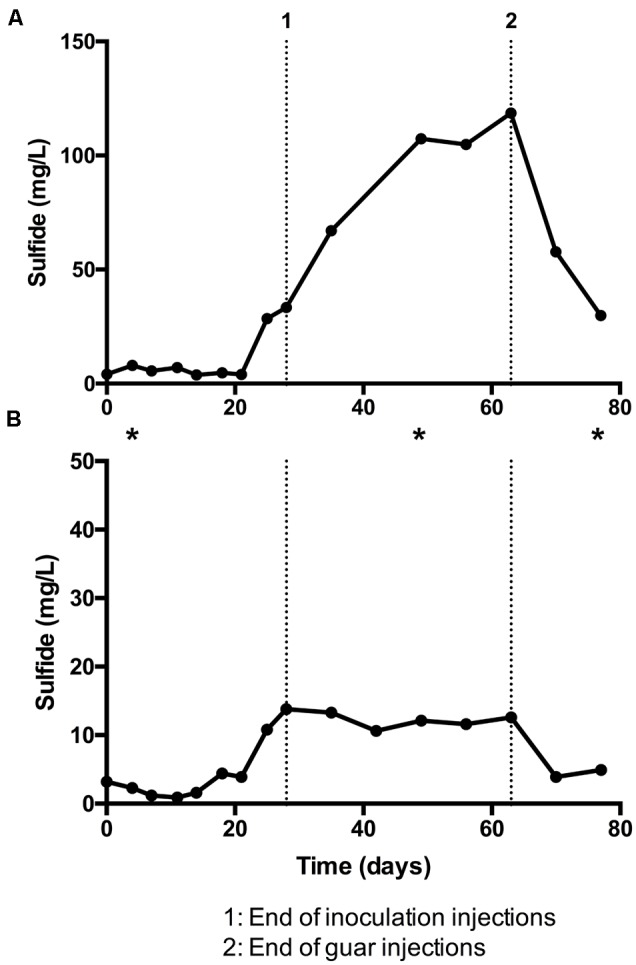
**Sulfide detected in guar gum bioreactor experiments, expressed as milligram per liter with time. (A)** Bioreactor 1, fed with sulfate-amended freshwater medium and packed with glass beads. **(B)** Bioreactor 2, fed with low-sulfate natural surface water and packed with sand. Asterisks denote timepoints at which samples were taken for DNA extraction and 16S rRNA sequencing.

Results from bioreactor experiments are summarized in **Figure 1**. Sulfide production was detected in both bioreactors. Much higher concentrations of sulfide were measured in bioreactor 1 (**Figure 1A**) compared to bioreactor 2 (**Figure 1B**). In both cases, the concentration of total sulfide was highest after inoculations had stopped, but before guar injection had ceased (**Figure 1**, points 1 and 2, respectively). In bioreactor 1, the average concentration of sulfide from day 0 to day 21 was 5.3 ± 1.6 mg/l. The average sulfide concentration for the same period in bioreactor 2 was 2.5 ± 1.4 mg/l. In both bioreactors the maximum total sulfide concentrations were recorded on day 63 (118.6 mg/l in bioreactor 1 and 12.6 mg/l in bioreactor 2). Sulfide concentrations decreased in both bioreactors after guar injections ceased. The average pH measured in bioreactors 1 and 2 was 8.3 and 7.6, respectively.

### Microbial Community Analysis

**FIGURE 2:**
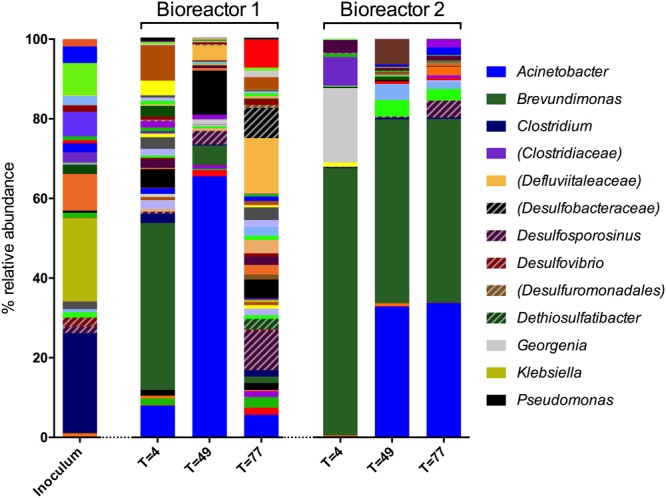
**Genus-level microbial community composition of bioreactor experiments after 4, 49, and 77 days based on 16S rRNA gene sequences.** Genera that constituted less than 1% combined abundance were omitted. Where genus level could not be resolved, the last matched taxonomic level of identification is given in parentheses. All patterned entries represent lineages implicated with sulfidogenesis. Included in the legend are lineages that represent 10% or more relative abundance in a sample, and lineages implicated with sulfidogenesis.

Microbial community composition was analyzed using 16S rRNA gene sequencing. A total of 281 OTUs were detected in the sulfate-reducing enrichment culture that was inoculated into both bioreactors (the inoculum). In bioreactor 1 samples, 599 OTUs were detected by day 4, 347 by day 49, and 420 by day 77. In bioreactor 2, 105 OTUs were observed by day 4, 135 by day 49, and 117 by day 77. Genus-level diversity of the inoculum and bioreactor samples is shown in **Figure 2**.

The inoculum was dominated by Firmicutes (64.0%, 78 OTUs), of which 58% of obtained sequences were affiliated to the class *Clostridium* (68 OTUs). The remainder of the sequences were affiliated with the phyla Proteobacteria (30.3%, 29 OTUs) and Bacteroidetes (5.5%, 9 OTUs). Other phyla accounted for less than 0.2% of the sequences from this sample. The most abundant genera detected in the inoculum were *Clostridium* (24.1%, 16 OTUs), *Klebsiella* (20.0%, 1 OTU) and *Sedimentibacter* (8.8%, 6 OTUs). Sequences of unidentified genera affiliated with the families Ruminococcaceae (7.8%, 13 OTUs), Enterobacteriaceae (5.9%, 1 OTU), Sphingobacteriaceae (4.0%, 1 OTU), and Clostridiaceae (2.4%, 2 OTUs) were also prominent. Sulfate-reducing genera detected in the inoculum include *Desulfovibrio* (2.2%, 1 OTU), *Desulfosporosinus* (1.5%, 1 OTU), and *Desulfitobacterium* (0.7%, 1 OTU), and 0.1% of sequences were assigned to the sulfur-reducing species *Geobacter sulfurreducens* (1 OTU), which is also a well known Fe(III)-reducing bacterium.

Sequences obtained from bioreactor 1 on day 4 were dominated by Proteobacteria (77.9%, 114 OTUs), most of which were affiliated to the class Alphaproteobacteria (50.1%, 28 OTUs), in addition to Gammaproteobacteria (15.2%, 20 OTUs) and Betaproteobacteria (10.1%, 46 OTUs). The most abundant genus in this sample was *Brevundimonas*, accounting for 32.2% of sequences (2 OTUs). Other prominent bacteria in this sample included members of the *Acinetobacter* (6.1%, 3 OTUs) and *Pseudomonas* (3.6%, 7 OTUs) genera. In contrast to the inoculum, sequences assigned to the *Clostridium* genus only accounted for 1.8% (7 OTUs) of the community at this time point.

By day 49, the bioreactor 1 community was dominated by Proteobacteria (83.3%, 102 OTUs). The dominant genera were *Acinetobacter* (63.2%, 3 OTUs), *Pseudomonas* (10.7%, 6 OTUs), and *Brevundimonas* (4.6%, 2 OTUs). By day 77, diversity of the community had substantially increased (see **Figure 2**). Roughly two-thirds of sequences were affiliated with Firmicutes (36.0%, 92 OTUs) and Proteobacteria (35.3%, 112 OTUs). The majority of sequences assigned to the former were identified as unknown members of the Defluviitaleaceae family, and 8.1% were assigned to the sulfate-reducing genus *Desulfosporosinus* (4 OTUs).

Bioreactor 2 was dominated with Proteobacteria throughout the experiment, accounting for 71.1% (41 OTUs), 85.6% (51 OTUs), and 80.7% (41 OTUs) of sequences by day 4, 49, and 77, respectively. Actinobacteria accounted for 18.5% of sequences at day 4 (11 OTUs), but less than 1% at the later time points. Firmicutes were less abundant than in the inoculum (64.0% 78 OTUs), but relatively stable throughout (9.4% at day 4, 18 OTUs; 7.3% at day 49, 29 OTUs; 9.8% at day 77, 29 OTUs). *Brevundimonas* was the dominant genus at every time point of the bioreactor 2 experiment, representing 65.0% of day 4 sequences (5 OTUs), 44.8% of those obtained by day 49 (4 OTUs), and 45.0% at day 77 (6 OTUs). Organisms affiliated with *Georgenia* species were also prominent by day 4, accounting for 18.2% of sequences (2 OTUs). Unknown members of the Clostridiaceae family represented 7.0% of sequences at the same time point (4 OTUs), but were not detected at later time points. Organisms most closely affiliated with *Acinetobacter* was the second-most abundant genus by day 49 (31.9%, 3 OTUs) and 77 (33.0%, 2 OTUs).

The dominant genera identified throughout both bioreactor experiments (representatives of *Brevundimonas, Acinetobacter*) were detected in the inoculum, albeit each accounted for less than 1% of the inoculum community. *Pseudomonas*, found to be relatively prominent in bioreactor 1, was also detected in the inoculum, although *Georgenia* (prominent in bioreactor 2) was not. The bioreactors were not sterile prior to use in these experiments, so may represent an additional source of microorganisms.

### Presence of Sulfidogenic Lineages

**FIGURE 3:**
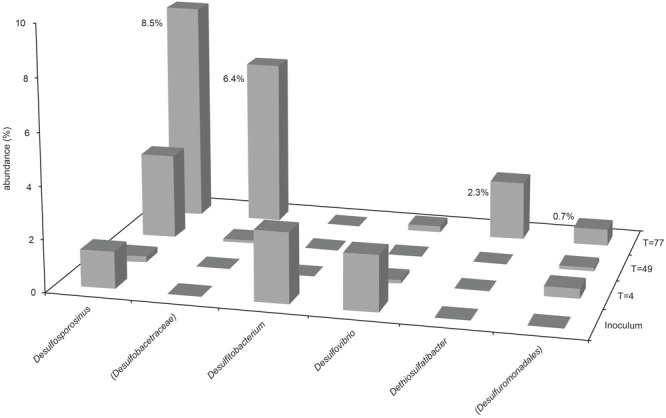
**Abundance of sulfidogenic taxa in Bioreactor 1 samples (sulfate-amended, packed with glass beads).** Where genus level could not be resolved, the last matched taxonomic level of identification is given in parentheses.

**FIGURE 4:**
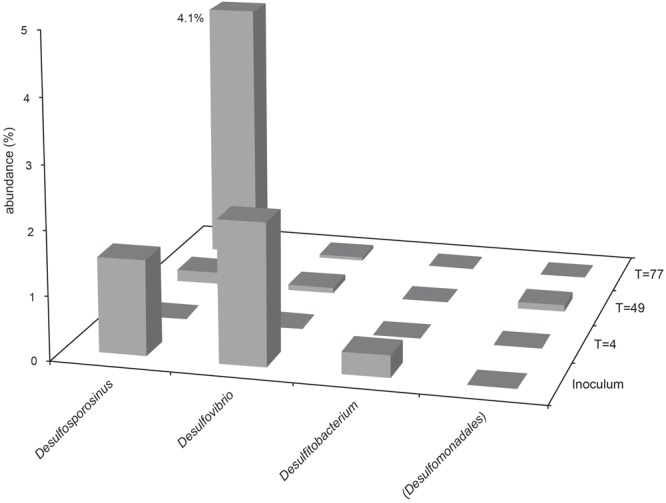
**Abundance of sulfidogenic taxa in Bioreactor 2 samples (low-sulfate, packed with sand).** Where genus level could not be resolved, the last matched taxonomic level of identification is given in parentheses.

The presence and abundance of genera associated with sulfide production are summarized in **Figures 3, 4**. Only assigned taxa that include known bacterial strains capable of sulfide production have been included in this analysis, however we acknowledge that other microorganisms may have contributed to sulfide production. The acetate oxidizing sulfate-reducing enrichment used to inoculate the bioreactors appeared to select for organisms most closely related to *Desulfovibrio* and *Desulfosporosinus* species, though only the latter appeared to increase in relative abundance in the bioreactor experiments (see **Figure 2**).

The most abundant sulfate-reducing genus in the inoculum was *Desulfovibrio*, although it only accounted for 2.4% of sequences (1 OTU) obtained from this sample, and less than 1% of the community in both bioreactor experiments at every time point. Close relatives of known *Desulfosporosinus* species were also present in the inoculum (1.6%, 3 OTUs), although less abundant than *Desulfovibrio* species. The relative abundance of *Desulfosporosinus* species increased in the later stages of the bioreactor 1 experiment, representing 10.2% (4 OTUs) of the day 77 community (see **Figures 2, 3**). The thiosulfate-reducing genus *Dethiosulfatibacter* was also detected in bioreactor 1 samples (2.7% of day 77 community, 1 OTU), but was not detected in the inoculum, presumably due to very low abundance, though its presence in the bioreactor prior to inoculation cannot be ruled out.

In bioreactor 2, *Desulfosporosinus* species were enriched by day 77, representing 4.1% of the community (1 OTU). *Desulfitobacterium* species were not detected in any samples from bioreactor 2. Organisms most closely affiliated with *Dethiosulfatibacter* and *Desulfobulbus* genera were both detected at day 4, but each accounted for less than 0.1% of the community, and were not detected in the inoculum or at later time points, either due to their low abundance in the inoculum or because they were already present in the bioreactor.

## Discussion

A number of organic additives are used in hydraulic fracturing fluid, and whether these additives stimulate deleterious microbial activity is poorly understood. The results from this study demonstrate that guar gum, a widely used gelling agent in disclosed hydraulic fracturing operations, can stimulate heterotrophic microorganisms including sulfate-reducing bacteria under conditions relevant to the terrestrial subsurface, even with low sulfate concentrations typical of surface water sources, ultimately fuelling biogenic sulfide production.

The enrichment of heterotrophic taxa in both bioreactor experiments (**Figure 2**) suggests that guar gum is readily bioavailable for microbial metabolism. Consistent with this was the detection of high concentrations of volatile fatty acids in both bioreactors at day 28 (principally acetate and propionate at 182.6 and 75.4 mg/l, respectively in bioreactor 1, and 169.4 mg/l acetate and 109.8 mg/l propionate in bioreactor 2), which depleted over the course of the experiment (data not shown). *Brevundimonas*, the most abundant genus detected early in the bioreactor 1 experiment and throughout the bioreactor 2 experiment (**Figure 2**), is a non-fermentative Gram-negative genus in the alpha subdivision of the Proteobacteria, able to draw upon a wide range of organic compounds for non-fermentative respiration and is known to produce organic acids as by-products (Segers et al., 1994). At least one strain of *Brevundimonas* is known to metabolize mannose and galactose, the two sugars that make up the guar gum galactomannan polysaccharide (Segers et al., 1994). It therefore seems likely that strains of *Brevundimonas* were able to metabolize guar gum and its potential degradation products in these experiments. This hypothesis warrants further investigation, including culturing experiments with and without guar. *Acinetobacter* was also prominent in later stages of both bioreactor experiments. Strains of this genus are similarly non-fermentative and metabolically versatile, capable of using an array of organic compounds as sole energy and carbon sources (Doughari et al., 2011). A large number of strains have been successfully isolated using acetate as the sole source of carbon and energy (Warskow and Juni, 1972), which may be present as a guar degradation product in the bioreactors. It is therefore feasible that strains of *Acinetobacter* are also able to use guar gum, directly or via its degradation products, in the experiments. Both genera were detected in the inoculum, and are assumed to originate from the reservoir water used to initiate the sulfate-reducing enrichment culture.

Unsurprisingly, sulfidogenic genera were more numerous and abundant in samples taken from bioreactor 1 (injected with sulfate-amended medium) compared with bioreactor 2 (injected with low-sulfate surface water). The most abundant sulfate-reducing genera in bioreactor 1 were *Desulfosporosinus, Dethiosulfatibacter* and unidentified members of the Desulfobacteraceae family (**Figures 2, 3**). Of these, only *Desulfosporosinus* was detected in the inoculum. It is possible that these other taxa were in fact present but in such small numbers that they were not detected during sequencing. Interestingly, *Dethiosulfatibacter* species are unable to reduce sulfate to sulfide, and instead utilize thiosulfate and elemental sulfur as electron acceptors (Takii et al., 2007), neither of which were supplied to the bioreactor. It is therefore likely that *Dethiosulfatibacter* strains were not contributing directly to sulfide production, and were instead operating a fermentative metabolism (Takii et al., 2007), contributing to the breakdown of guar gum. Some sulfate-reducing genera that were enriched in the inoculum did not prosper in the bioreactor experiment, such as *Desulfitobacterium* and *Desulfovibrio* (**Figure 3**). The data therefore suggest that *Desulfosporosinus* and members of the sulfate-reducing family Desulfobacteraceae were responsible for sulfide production measured in the bioreactor 1 experiment. The sulfide production measured in bioreactor 2 (**Figure 1**) can most likely be attributed to *Desulfosporosinus*, the only sulfidogenic taxon that increased in abundance throughout the course of the experiment (**Figure 4**). In both cases it is possible that taxa other than those discussed here may have contributed to sulfide production.

The sulfide production measured in both bioreactors does not appear to be limited by the amount of sulfate available. Based on a 1:1 ratio of sulfate reduced to sulfide produced (whether direct or via intermediate sulfur species), the maximum amount of sulfide that could be produced is equal to the concentration of sulfate available in each bioreactor, assuming the supply electron donors is not limiting. Therefore, 15.7 mM sulfate added to bioreactor 1 has the potential liberate 15.7 mM (519 mg/l) sulfide, yet the maximum concentration measured was almost five times lower. Similarly, only 12.6 mg/l total sulfide was measured in bioreactor 2, more than six times lower than the maximum concentration that could be produced from the direct reduction of 79.6 mg/l sulfate measured in the pond water. In both cases it therefore appears that the supply of electron donors was limiting.

### Results Compared to *In Situ* Studies of Shale Gas Plays

A number of other studies have identified sulfidogenic microorganisms obtained from active shale gas plays in the US. Struchtemeyer and Elshahed (2012) conducted 16S rRNA diversity analysis on flowback fluids from shale gas wells in the Barnett shale, and identified sequences affiliated with the sulfate-reducing genera *Desulfosporosinus, Desulfotomaculum*, as well as thiosulfate- and sulfur-reducing genera *Dethiosulfovibrio, Thermotoga, Petrotoga, Thermovirga*, and *Halanaerobium*. Davis et al. (2012) monitored the change in microbial communities in post-fracturing fluids stored in tanks in the Barnett shale formation over a 6-month period using 16S rRNA gene sequencing, and found the number of sequences affiliated with *Desulfovibrio* increased over time. However, their results indicate that the thiosulfate-reducing *Halanaerobium* genus was more abundant, and likely contributed to the biogenic sulfide production reported in this area (Fichter et al., 2008, 2009). Indeed, it is common that microorganisms other than those traditionally thought of as sulfate-reducing bacteria are the dominant sulfide-producers in conventional hydrocarbon reservoirs, especially at elevated temperature and pressure (Gittel et al., 2009; Stevenson et al., 2011) *Desulfovibrio* was found to be abundant in flowback fluids from one well in the Antrim shale gas play (Kirk et al., 2012), consistent with results reported by Davis et al. (2012). *Desulfobacter halotolerans* was abundant in a flowback impoundment in the Marcellus shale, despite treatment with biocide, though *Halanaerobium congolense* accounted for more than half of sequences obtained from 16S rRNA analysis (Mohan et al., 2013b). *Halanaerobium* is a genus of halophilic bacteria, capable of fermentation of wide array of organics as well as thiosulfate- and sulfur-reduction (Zeikus et al., 1983), and was also found to dominate sequences from produced waters in other studies of the same shale gas play (Mohan et al., 2013a; Cluff et al., 2014; Daly et al., 2016). Strains of the same genus in flowback fluids were later shown to be viable (Akob et al., 2015), and, more recently, capable of degrading guar gum (Liang et al., 2016).

It is clear that sulfidogenic taxa are common in flowback and produced fluids from active shale gas plays, though the overlap with those identified in our experiments is limited to *Desulfosporosinus* species (Struchtemeyer and Elshahed, 2012). In addition, a number of studies have identified the prominence of non-sulfate-reducing sulfidogenic taxa, especially stains of *Halanaerobium*. This genus was absent from our bioreactor samples, in part owing to the lack of thiosulfate and high salt concentrations in the systems.

### Bioreactor Compared with *In Situ* Conditions

In this study, we sought to simulate conditions more relevant to the deep terrestrial subsurface compared with conventional serum bottle substrate utilization tests. In particular, bioreactor experiments were run at high pressure (1000 psi, 68.9 bar, 6.89 MPa), and bioreactor 2 was fed with terrestrial surface water which represents a plausible source of fracturing fluid water in future UK shale gas extraction (CIWEM, 2016). Furthermore, the sulfate-reducing enrichment culture was initiated with water from a drinking water reservoir, a similarly plausible source of water for UK hydraulic fracturing operations, and thus the microbial community introduced to the bioreactor experiments could be considered highly appropriate.

A number of parameters at play in the hydraulic fracturing of shale formations were not, however, represented in the experiments reported here. These parameters are likely to impact on bacterial community composition, and can account for the major differences in our study compared with *in situ* studies. For example, hydraulic fracturing leads to significant changes in fracturing fluid composition that were not reflected in the bioreactor experiments. During shale gas extraction, input fluids are subjected to temperatures above 50°C, and pressures greater than 30 MPa (Fichter et al., 2012; Picard and Daniel, 2013). Upon contact with freshly fractured shale, these fluids are influenced by the chemical composition of the formation, observed in flowback and produced waters as high concentrations of total dissolved solids, dissolved organic carbon, naturally-occurring radioactive minerals, lower pH, and salinities that can reach several times that of seawater (Struchtemeyer and Elshahed, 2012; Mohan et al., 2013a; Akob et al., 2015; Liang et al., 2016). Furthermore, the increased temperatures encountered by hydraulic fracturing fluids at depth may alter the properties (and hence the bioavailability) of organic additives, including guar gum. Significant changes in microbial diversity can be seen as a result of these well-documented chemical changes, from a typical freshwater aerobic community in input fluids, to a less diverse anaerobic community in flowback fluids. The predominance of halotolerant and halophilic taxa in flowback waters indicates that salinity is a significant contributing factor to changes in microbial ecology (Struchtemeyer and Elshahed, 2012; Mohan et al., 2013a; Cluff et al., 2014; Akob et al., 2015; Daly et al., 2016), and most likely accounts for the lack of halophilic sulfidogens, such as *Halanaerobium* (Zeikus et al., 1983), in our experiments. It is worth noting, however, that relatively few shale gas plays have been assessed with regard to microbial activity, and it remains unclear to what extent the predominance of certain taxa over others are controlled by factors such as the choice of source water used for fracturing fluids, the characteristics of the shale formations, the mix of additive used, and operational parameters chosen. These factors may change significantly in shale gas extraction development outside of the US. The experiments reported here thus serve as a blueprint for an *ex situ* method superior to simple microcosm tests. This methodology can be applied extensively to better constrain the factors that contribute to souring and other deleterious microbial activities during shale gas extraction operations.

### Sources of Carbon and Energy for Biogenic Sulfide Production during Shale Gas Extraction

In this study, we have demonstrated that guar gum, a commonly used gelling agent added to fracturing fluids, has the potential to stimulate biogenic sulfide production. However guar gum is not the only potential electron donor for microbial activity during shale gas extraction. An alternative gelling agent to guar is cellulose, readily fermented by heterotrophic bacteria (e.g., Weimer and Zeikus, 1977), the by-products of which could serve as electron donors for sulfidogenesis. Acetate is a well-known electron donor for microbial sulfate reduction (e.g., Laanbroek and Pfennig, 1981), and is added to fracturing fluids to control pH (Elsner and Hoelzer, 2016). It is also plausible that other fracturing fluid additives known to be bioavailable to microorganisms, such as polyacrylamide (Nakamiya and Kinoshita, 1995; Wen et al., 2010) and ethylene glycol (Haines and Alexander, 1975; Watson and Jones, 1977; McGahey and Bouwer, 1992), would be broken down into directly available electron donors during hydraulic fracturing.

Another source of organic carbon and energy is the formation itself. Shale is organic rich, and whilst its nanodarcy permeability is thought to render it uninhabitable, the organic compounds they host are thought to support microbial communities at the interface of more permeable strata (Frederickson et al., 1997; Krumholz et al., 2002). It is clear from the organic chemistry of flowback and produced fluids that hydraulic fracturing liberates hydrocarbons from the formation (Strong et al., 2014). These hydrocarbons could conceivably serve as an indirect source of electron donors after undergoing fermentation by heterotrophic bacteria (e.g., species of *Halanaerobium*, Zeikus et al., 1983). Additionally, highly oxidizing peroxide compounds are added to fracturing fluids to reduce fluid viscosity and allow for fluid recovery prior to gas flow, and these compounds may render otherwise recalcitrant kerogen more bioavailable through oxidation.

Struchtemeyer et al. (2011) demonstrated that organic polymers in drilling waters were degraded by a community of anaerobic microorganisms present in drilling muds. Barite (BaSO_4_) and sulfonates, added to drilling mud to add weight and reduce viscosity, respectively, were also found to stimulate sulfate-reducing microorganisms and biogenic sulfide production (Struchtemeyer et al., 2011). The act of drilling into shale formations prior to hydraulic fracturing can therefore introduce additional carbon and energy sources for sulfidogenesis before organic-rich fracturing fluids are injected.

Additional sources of sulfur compounds can also be found in injection fluids, though to a much lesser extent than organic carbon. In a thorough review of disclosed fracturing fluid additives, Elsner and Hoelzer (2016) report the use of organo sulfonates as surfactants in 4% of operations, sodium sulfate to control ionic strength in 2.4% of operations, ammonium sulfate as a friction reducer in 1.1%, and ferric sulfate as a crosslinker in less than 1%. The source of water used to make up fracturing fluid may also harbor sulfate concentrations sufficient to lead to detectable biogenic sulfide production, though this will vary with operation location. In addition, it is plausible that oxidizing agents added to fracturing fluids to reduce viscosity for fluid recovery (breakers) could oxidize any reduced sulfur native to the formation [either as pyrite (Raiswell and Berner, 1986; Gross et al., 2015) or organo-sulfur compounds (Tissot and Welte, 1978; Sinninghe Damsté and de Leeuw, 1989)], thereby rendering it available to support sulfidogenesis through sulfate-, thiosulfate-, or sulfur-reduction.

## Conclusion

Using bespoke high-pressure bioreactor systems, we have demonstrated that guar gum, the most commonly used gelling agent in hydraulic fracturing fluids, can serve as a carbon and electron donor source for a sulfate-reducing microbial community, leading to biogenic sulfide production. These results have important implications for our understanding of the critical factors controlling souring during shale gas extraction, and highlight the value of adopting a bespoke bioreactor methodology to study these constraints in isolation. Future research should seek to accurately simulate the elevated pressures, temperatures and aqueous geochemistry encountered during shale gas extraction. This approach could be used to assess the potential for other additives to stimulate sulfidogenesis and other microbial processes, for example, biofilm formation and clogging, using the pressurized bioreactor model employed here, in order to better define the least problematic fracturing fluid composition, and to identify the microorganisms that need to be controlled prior to re-use of flowback fluids. The methodology adopted in this study offers the opportunity to probe the microbial metabolisms observed recently to be active in hydraulic fracturing produced waters in more detail (Daly et al., 2016). In particular, our approach allows for the relative influence of fracturing fluid additives on the activity of these metabolisms to be constrained beyond what is possible through *in situ* studies.

## Author Contributions

SN, BE, KT, and JL designed the experiments. SN, LW, and MS conducted the experiments. CB carried out DNA extractions and sequencing. SN carried out data analysis, prepared figures, and wrote the manuscript with contributions from all authors.

## Conflict of Interest Statement

The authors declare that the research was conducted in the absence of any commercial or financial relationships that could be construed as a potential conflict of interest.

## Footnotes

1 http://www.bioinformatics.babraham.ac.uk/projects/fastqc/

2 http://www.ncbi.nlm.nih.gov/sra/

## References

[B1] Akob D. M. Cozzarelli I. M. Dunlap D. S. Rowan E. L. Lorah M. M. (2015). Organic and inorganic composition and microbiology of produced waters from Pennsylvania shale gas wells. *Appl. Geochem.* 60 116–125. 10.1016/j.apgeochem.2015.04.011

[B2] Andrews I. J. (2013). *The Carboniferous Bowland Shale Gas Study: Geology and Resource Estimation*. London: British Geological Survey for Department of Energy and Climate Change 64.

[B3] Caporaso J. G. Kuczynski J. Stombaugh J. Bittinger K. Bushman F. D. Costello E. K. (2010). QIIME allows analysis of high-throughput community sequencing data. *Nat. Methods* 7 335–336. 10.1038/nmeth.f.303 20383131 PMC3156573

[B4] Caporaso J. G. Lauber C. L. Walters W. A. Berg-Lyons D. Huntley J. Fierer N. (2012). Ultra-high-throughput microbial community analysis on the Illumina HiSeq and MiSeq platforms. *ISME J.* 6 1621–1624. 10.1038/ismej.2012.8 22402401 PMC3400413

[B5] Caporaso J. G. Lauber C. L. Walters W. A. Berg-Lyons D. Lozupone C. A. Turnbaugh P. J. (2011). Global patterns of 16S rRNA diversity at a depth of millions of sequences per sample. *Proc. Natl. Acad. Sci. U.S.A.* 108(Suppl. 1) 4516–4522. 10.1073/pnas.1000080107/-/DCSupplemental 20534432 PMC3063599

[B6] CIWEM (2016). *Shale Gas and Water: An Independent Review of Shale Gas Extraction in the UK and the Implications for the Water Environment*. London: Chartered Institution of Water and Environmental Management (CIWEM).

[B7] Cluff M. A. Hartsock A. MacRae J. D. Carter K. Mouser P. J. (2014). Temporal changes in microbial ecology and geochemistry in produced water from hydraulically fractured Marcellus shale gas wells. *Environ. Sci. Technol.* 48 6508–6517. 10.1021/es501183p 24803059

[B8] Crociani F. Alessandrini A. Mucci M. M. Biavati B. (1994). Degradation of complex carbohydrates by *Bifidobacterium* spp. *Int. J. Food Microbiol.* 24 199–210. 10.1016/0168-1605(94)90119-8 7703014

[B9] Daly R. A. Borton M. A. Wilkins M. J. Hoyt D. W. Kountz D. J. Wolfe R. A. (2016). Microbial metabolisms in a 2.5-km-deep ecosystem created by hydraulic fracturing in shales. *Nat. Microbiol.* 10.1038/nmicrobiol.2016.146 [Epub ahead of print]. 27595198

[B10] Davis J. P. Struchtemeyer C. G. Elshahed M. S. (2012). Bacterial communities associated with production facilities of two newly drilled thermogenic gas wells in the Barnett Shale (Texas, USA). *Environ. Microbiol.* 64 942–954. 10.1007/s00248-012-0073-3 22622766

[B11] Doughari H. J. Ndakidemi P. A. Human I. S. Benade S. (2011). The ecology, biology and pathogenesis of *Acinetobacter* spp.: an overview. *Microbes Environ.* 26 1–12. 10.1264/jsme2.ME10179 21502736

[B12] Eden B. Laycock P. J. Fielder M. (1993). *Oilfield Reservoir Souring.* Sudbury: HSE Books.

[B13] Edgar R. C. (2010). Search and clustering orders of magnitude faster than BLAST. *Bioinformatics* 26 2460–2461. 10.1093/bioinformatics/btq461 20709691

[B14] Edgar R. C. (2013). UPARSE: highly accurate OTU sequences from microbial amplicon reads. *Nat. Methods* 10 996–998. 10.1038/nmeth.2604 23955772

[B15] Elsner M. Hoelzer K. (2016). Quantitative survey and structural classification of hyadrulic fracturing chemicals reported in unconventional gas production. *Environ. Sci. Technol.* 50 3290–3314. 10.1021/asc.est.5b02818 26902161

[B16] Fichter J. Moore R. Braman S. Wunch K. Summer E. Holmes P. (2012). “How hot is too hot for Bacteria? A technical study assessing bacterial establishment in downhole drilling, fracturing and stimulation operations,” in *Proceedings of the NACE International Conference and Expo, March* 11–15 Salt Lake City, UT.

[B17] Fichter K. Johnson K. French K. Oden R. (2008). “Use of microbiocides in Barnett shale gas well fracturing fluids to control bacterially-related problems,” in *Proceedings of the NACE International Conference and Expo, Paper 08658 March* 16–19 New Orleans, LA.

[B18] Fichter K. Johnson K. French K. Oden R. (2009). Biocides control Barnett Shale fracturing fluid contamination. *Oil Gas J.* 107 38–44.

[B19] Fonselius S. H. Dryssen D. Yhlen B. (1999). “Determination of hydrogen sulphide,” in *Methods of Seawater Analysis* 3rd Edn eds Grasshoff K. Kremling K. Ehrhardt M. (New York, NY: Wiley) 91–100.

[B20] Frederickson J. K. McKinley J. P. Bjornstad B. N. Long P. E. Ringelberg D. B. White D. C. (1997). Pore-size constraints on the activity and survival of subsurface bacteria in the late cretaceous shale-sandstone sequence, northwestern New Mexico. *Geomicrobiol. J.* 14 183–202. 10.1080/01490459709378043

[B21] Gittel A. Sørensen K. B. Skovhus T. L. Ingvorsen K. Schramm A. (2009). Prokaryotic community structure and sulfate reducer activity in water from high-temperature oil reservoirs with and without nitrate treatment. *Appl. Environ. Microbiol.* 75 7086–7096. 10.1128/AEM.01123-09 19801479 PMC2786513

[B22] Gross D. Sachsenhofer R. F. Bechtel A. Pytlak L. Rupprecht B. Wegerer E. (2015). Organic geochemistry of Mississippian shales (Bowland Shale Formation) in central Britain: implications for depositional environment, source rock and gas shale potential. *Mar. Pet. Geol.* 59 1–21. 10.1016/jmarpetgeo.2014.07.022

[B23] Haas B. J. Gevers D. Earl A. M. Feldgarden M. Ward D. V. Giannoukos G. (2011). Chimeric 16S rRNA sequence formation and detection in Sanger and 454-pyrosequenced PCR amplicons. *Genome Res.* 21 494–504. 10.1101/gr.112730.110 21212162 PMC3044863

[B24] Haines J. R. Alexander M. (1975). Microbial degradation of polyethylene glycols. *Appl. Environ. Microbiol.* 29 621–625. 10.1128/am.29.5.621-625.1975 PMC187047 807161

[B25] Joshi N. A. Fass J. N. (2011). *Sickle: A Sliding-Window, Adaptive, Quality-Based Trimming Tool for FastQ Files (Version 1.33) [Software].* Available at https://github.com/najoshi/sickle

[B26] Kirk M. F. Martinin A. M. Breecker D. O. Colman D. R. Takacs-Vesback C. Petsch S. T. (2012). Impact of commercial natural gas production on geochemistry and microbiology in a shale-gas reservoir. *Chem. Geol.* 33 15–25. 10.1016/j.chemgeo.2012.08.032

[B27] Kozich J. J. Westcott S. L. Baxter N. T. Highlander S. K. Schloss P. D. (2013). Development of a dual-index sequencing strategy and curation pipeline for analyzing amplicon sequence data on the MiSeq illumina sequencing platform. *Appl. Environ. Microbiol.* 79 5112–5120. 10.1128/AEM.01043-13 23793624 PMC3753973

[B28] Krumholz L. R. Harris S. H. Suflita J. M. (2002). Anaerobic microbial growth from components of Cretaceous Shales. *Geomicrobiol. J.* 19 593–602. 10.1080/01490450290098559

[B29] Laanbroek H. J. Pfennig N. (1981). Oxidation of short-chain fatty acids by sulfate-reducing bacteria in freshwater and in marine sediments. *Arch. Microbiol.* 128 330–335. 10.1007/BF00422540 7212933

[B30] Liang R. Davidova I. A. Marks C. R. Stamps B. W. Harriman B. H. Stevenson B. S. (2016). Metabolic capability of a predominant *Halanaerobium* sp. in hydraulically fractured gas wells and its implication in pipeline corrosion. *Front. Microbiol.* 7:988 10.3389/fmicb.2016.00988 PMC4916785 27446028

[B31] Martin M. (2011). Cutadapt removes adapter sequences from high-throughput sequencing reads. *EMBnet J.* 17 10–12. 10.14806/ej.17.1.200

[B32] Masella A. P. Bartram A. K. Truszkowski J. M. Brown D. G. Neufeld J. D. (2012). PANDAseq: paired-end assembler for illumina sequences. *BMC Bioinformatics* 13:31 10.1186/1471-2105-13-31 PMC3471323 22333067

[B33] McGahey C. Bouwer E. J. (1992). Biodegradation of ethylene glycol in simulated subsurface environments. *Water Sci. Technol.* 26 41–49.

[B34] Mohan A. M. Hartsock A. Bibby K. J. Hammack R. W. Vidic R. D. Gregory K. B. (2013a). Microbial community changes in hydraulic fracturing fluids and produced water from shale gas extraction. *Environ. Sci. Technol.* 47 13141–13150. 10.1021/es402928b 24088205

[B35] Mohan A. M. Hartsock A. Hammack R. W. Vidic R. D. Gregory K. B. (2013b). Microbial communities in flowback water impoundments from hydraulic fracturing for recovery of shale gas. *FEMS Microbiol. Ecol.* 86 567–580. 10.1111/1574-6941.12183 23875618

[B36] Nakamiya K. Kinoshita S. (1995). Isolation of polyacrylamide-degrading bacteria. *J. Ferment. Bioeng.* 80 418–420. 10.1111/1574-6941.12183

[B37] Nurk S. Bankevich A. Antipov D. Gurevvich A. Korobeynikov A. Lapidus A. (2013). “Assembling genomes and mini-metagenomes from highly chimeric reads,” in *Proceedings of the 17th Annual International Conference, RECOMB 2013: Research in Computational Molecular Biology, Beijing, China, April 7-10 2013* eds Deng M. Jiang R. Sun F. Zhang X. (Berlin: Springer) 158–170.

[B38] Picard A. Daniel I. (2013). Pressure as an environmental parameter for microbial life – a review. *Biophys. Chem.* 183 30–41. 10.1016/j/bpc.2013.06.019 23891571

[B39] Raiswell R. Berner R. A. (1986). Pyrite and organic matter in Phanerozoic normal marine shales. *Geochim. Cosmochim. Acta* 50 1967–1976. 10.1016/0016-7037(86)90252-8

[B40] Schink B. (1984). *Clostridium magnum* sp. nov., a non-autotrophic homoacetogenic bacterium. *Arch. Microbiol.* 137 250–255. 10.1007/BF00414553

[B41] Segers P. Vancanneyt M. Pot B. Torck U. Hoste B. Dewettinck D. (1994). Classification of *Pseudomonas diminuta* Leifson and Hugh 1954 and *Pseudomonas vesicularis* Büsing, Döll, and Freytag 1953 in *Brevundimonas* gen. nov. as *Brevundimonas diminuta* comb. nov. and *Brevundimonas vesicularis* comb. nov., respectively. *Int. J. Syst. Bacteriol.* 44 499–510. 10.1099/00207713-44-3-499 8068543

[B42] Sinninghe Damsté J. S. de Leeuw J. W. (1989). Analysis, structure and geochemical significance of organically-bound sulphur in the geosphere: state of the art and future research. *Org. Geochem.* 16 1077–1101. 10.1016/0146-6380(90)90145-P

[B43] Stevenson B. S. Drilling H. S. Lawson P. A. Duncan K. E. Parisi V. A. Suflita J. M. (2011). Microbial communities in bulk fluids and biofilms of an oil facility have similar composition but different structure. *Environ. Microbiol.* 13 1078–1090. 10.1111/j.1462-2920.2010.02413.x 21261797

[B44] Strong L. C. Gould T. Kasinkas L. Sadowsky M. J. Aksan A. Wackett L. P. (2014). Biodegradation in waters from hydraulic fracturing: chemistry, microbiology, and engineering. *J. Environ. Eng.* 140:B4013001 10.1061/(ASCE)EE.1943-7870.0000792

[B45] Struchtemeyer C. G. Davis J. P. Elshahed M. S. (2011). Influence of the drilling mud formulation process on the bacterial communities in thermogenic natural gas wells of the Barnett Shale. *Appl. Environ. Microbiol.* 77 4744–4753. 10.1128/AEM.00233-11 21602366 PMC3147393

[B46] Struchtemeyer C. G. Elshahed M. S. (2012). Bacterial communities associated with hydraulic fracturing fluids in thermogenic natural gas wells in North Central Texas, USA. *FEMS Microbiol. Ecol.* 81 13–25. 10.1111/j.1574-6941.2011.01196.x 22066833

[B47] Takii S. Hanada S. Tamaki H. Ueno Y. Sekiguchi Y. Ibe A. (2007). *Dethiosulfatibacter aminovorans* gen. nov., sp. nov., a novel thiosulfate-reducing bacterium isolated from coastal marine sediment via sulfate-reducing enrichment with Casamino acids. *Int. J. Syst. Evol. Microbiol.* 57 2320–2326. 10.1099/ijs.0.64882-0 17911304

[B48] Tanner R. S. (1989). Monitoring sulfate-reducing bacteria: comparison of enumeration media. *J. Microbiol. Methods* 10 83–90. 10.1016/0167-7012(89)90004-3

[B49] Tissot B. Welte D. H. (1978). *Petroleum Formation and Occurrence.* Berlin: Springer.

[B50] Tomlin J. Read N. W. Edwards C. A. Duerden B. I. (1986). The degradation of guar gum by a faecal incubation system. *Br. J. Nutr.* 55 481–486. 10.1079/BJN19860055 3676171

[B51] Walther R. Hippe H. Gottschalk G. (1977). Citrate, a specific substrate for the isolation of *Clostridium sphenoides*. *Appl. Environ. Microbiol.* 33 955–962. 869540 10.1128/aem.33.4.955-962.1977 PMC170796

[B52] Wang Q. Garrity G. M. Tiedje J. M. Cole J. R. (2007). Naïve Bayesian classifier for rapid assignment of rRNA sequences into the new bacterial taxonomy. *Appl. Environ. Microbiol.* 73 5261–5267. 10.1128/AEM.00062-07 17586664 PMC1950982

[B53] Warskow A. Juni E. (1972). Nutritional requirements of *Acinetobacter* strains isolated from soil, water and sewage. *J. Bacteriol.* 112 1014–1016. 4563966 10.1128/jb.112.2.1014-1016.1972 PMC251516

[B54] Watson G. K. Jones N. (1977). The biodegradation of polyethylene glycols by sewage bacteria. *Water Res.* 11 95–100. 10.1016/0043-1354(77)90189-0

[B55] Weijermars R. (2013). Economic appraisal for shale gas plays in Continental Europe. *Appl. Energy* 106 100–115. 10.1016/j.apenergy.2013.01.025

[B56] Weimer P. J. Zeikus J. G. (1977). Fermentation of cellulose and cellobiose by *Clostridium thermocellum* in the absence of *Methanobacterium thermoautotrophicum*. *Appl. Environ. Microbiol.* 33 289–297. 848953 10.1128/aem.33.2.289-297.1977 PMC170680

[B57] Wen Q. Chen Z. Zhao Y. Zhang H. Feng Y. (2010). Biodegradation of polyacrylamide by bacteria isolated from activated sludge and oil-contaminated soil. *J. Hazard. Mater.* 175 955–959. 10.1016/j.hazmat.2009.10.102 19932560

[B58] Zeikus J. G. Hegge P. W. Thompson T. E. Phelps T. J. Langworthy T. A. (1983). Isolation and description of *Haloanaerobium prevalens* gen. nov. and sp. nov., an obligate halophile common to Great Salt Lake sediments. *Curr. Microbiol.* 9 225–233. 10.1007/BF01567586

